# Hydroa Vacciniforme Lymphoproliferative Disorder With a Truncating KLF2 Variant Presenting as Benign Photodermatoses

**DOI:** 10.1111/phpp.70096

**Published:** 2026-04-29

**Authors:** John Warner‐Levy, Angelia Ong, Nina Farquharson, Jean Ayer

**Affiliations:** ^1^ Faculty of Biology, Medicine and Health The University of Manchester Manchester UK; ^2^ Department of Dermatology, Cutaneous Biology Research Center Massachusetts General Hospital and Harvard Medical School Boston Massachusetts USA; ^3^ Department of Histopathology, the Christie NHS Foundation Trust Manchester UK; ^4^ The Dermatology Centre, Salford Royal Hospital, Northern Care Alliance NHS Foundation Trust Salford UK; ^5^ Photobiology Unit, Dermatology Centre Salford Royal Hospital, Northern Care Alliance NHS Foundation Trust, Manchester Academic Health Science Centre Salford Greater Manchester UK

**Keywords:** EBV, Epstein–Barr virus, HVLPD, Hydroa Vacciniforme lymphoproliferative disorder, KLF2, photosensitivity

## 1 Background

Hydroa vacciniforme lymphoproliferative disorder (HVLPD) comprises a rare spectrum of EBV‐positive T‐ and NK‐cell lymphoid proliferations and lymphomas of childhood [1]. A ‘classic’ form is characterized by cutaneous‐limited disease with a clinically indolent course and is most frequently reported in White populations. In contrast, a ‘systemic’ form is more frequently observed in Asian and Latin American cohorts and is characterised by extracutaneous involvement with an increased risk of lymphomatous progression [2, 3].

We report an exceptionally rare case of HVLPD with late‐adolescent onset, initially diagnosed as polymorphic light eruption and later provisionally as actinic prurigo. This case emphasises that phenotypic overlap with benign photodermatoses may obscure timely recognition of a lymphoproliferative disorder with potential for lymphomatous transformation.

## 2 Case

A 30‐year‐old white woman was referred for evaluation of a 13‐year history of photosensitivity. Her initial episode occurred during vacation as a pruritic, erythematous eruption on the forearms, resolving with residual scaling and recurring on subsequent vacations with increasing severity. By age 22, episodes progressed to vesiculobullous lesions involving the legs and feet and were diagnosed as polymorphic light eruption following emergency department assessment. In April 2025, an episode in the United Kingdom was characterised by ulcerated plaques involving the arms, shoulders, lips, ears, nasal tip and scalp, with secondary impetiginisation and scarring. She denied constitutional symptoms but reported a temporal association with respiratory tract infections. Medical history included allergic rhinitis and childhood atopic dermatitis, managed with fexofenadine. There was no family history of atopy or photosensitivity. She reported regular use of broad‐spectrum sunscreen and underwent photodermatologic evaluation in July 2025.

Examination revealed no active eruption. Dermatology Life Quality Index scores were 5 in the preceding week and 21 over the preceding year. Laboratory investigations showed negative connective tissue and plasma porphyrin screens, with normal vitamin D and IgE levels. HLA typing demonstrated HLA‐DRB1*04:07 positivity, and EBV PCR detected viraemia (22,918 IU/mL). EBV serology was compatible with either EBV reactivation or a recent primary infection with waning VCA IgM.

Monochromator testing across narrow bandwidths of UVB, UVA and visible light demonstrated normal minimal erythema doses (MEDs). In contrast, broadband UVA provocation at 15 J/cm^2^ on the right arm produced moderate confluent erythema immediately and at 24 h. Solar simulator testing at 10 J/cm^2^ on the left arm induced faint blotchy erythema immediately, progressing to mild blotchy erythema at 24 h. Identical provocation of the left ear produced no immediate response but resulted in mild oedema and erythema at 24 h, whereas testing of the left nasal bridge caused immediate erythema evolving into an erythematous plaque at 24 h. Patch testing and photopatch testing for sunscreen filters and NSAIDs were negative. Considering the chronic pruritic photosensitivity and the presence of HLA‐DRB1*04:07, a provisional diagnosis of actinic prurigo was considered [4]. She was prescribed mometasone furoate ointment for the body and betamethasone valerate ointment for the face during flares.

A punch biopsy of the left ear demonstrated epidermal necrosis with ulceration. A dense inflammatory infiltrate was present in the reticular dermis and subcutaneous fat, with focal extension into the oedematous papillary dermis. The infiltrate comprised lymphoid cells and histiocytes with lesser neutrophils, eosinophils and plasma cells. Immunohistochemistry showed a predominantly T‐cell infiltrate (positive for BF‐1, CD2, CD3, CD5 and CD7, and negative for CD56), with a CD4:CD8 ratio of 1.5–2:1. EBER in situ hybridisation (EBER‐ISH) highlighted numerous EBV‐positive lesional T cells, with LMP expression in a smaller subset. T‐cell receptor gene rearrangement studies demonstrated clonal TRG and TRB peaks. The patient was referred to the supraregional cutaneous lymphoma service and discussed at a cutaneous lymphoma multidisciplinary meeting. EBV‐positive lesional T cells, clonal T‐cell receptor gene rearrangement and EBV viraemia, together with the clinical features, were highly suggestive of HVLPD.

**FIGURE 1:**
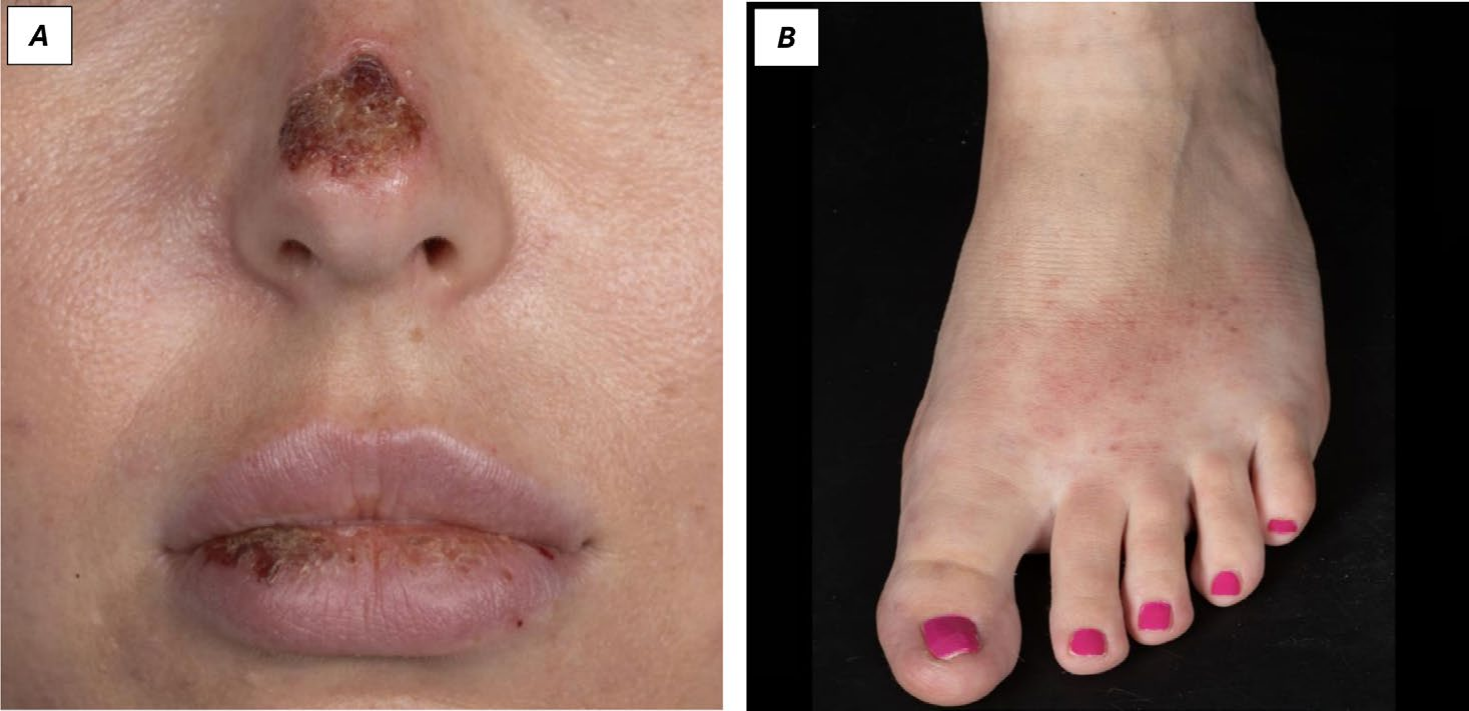
(A) Ulcerated nasal bridge and lower lip. (B) Eczematous lesions of the foot.

**FIGURE 2:**
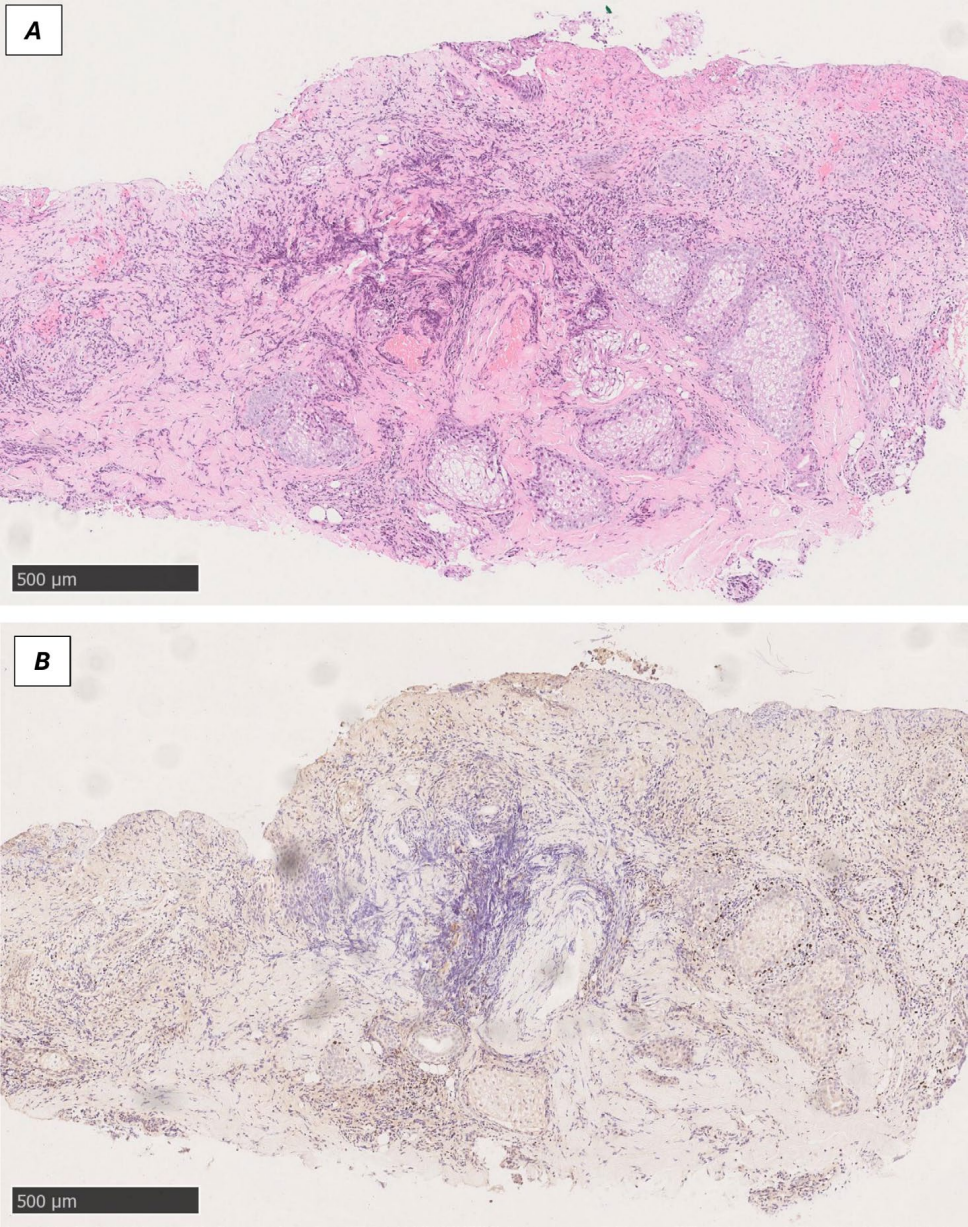
(A) H&E‐stained section of nasal bridge punch biopsy. (B) EBER in situ hybridisation of nasal bridge punch biopsy (scale bar = 500 μm).

In September 2025, the patient experienced a further flare while on vacation. Examination revealed an ulcerated nasal bridge with marked erythema and severe cheilitis. Blistering predominantly affected the lower lip, with scaling and inflammation of the bilateral pinnae. Shoulder nodules were noted, and plantar lesions demonstrated an eczematous morphology (Figure 1). A second biopsy, performed on the nasal bridge, demonstrated similar features to the previous biopsy, with extensive surface ulceration overlying a fibrotic dermis (Figure 2). A mixed infiltrate of lymphoid cells and histiocytes with rare neutrophils was present. There was no significant epidermotropism. The lymphoid cells were predominantly T cells, showing expression of pan‐T‐cell markers with partial loss of CD7, CD56 negativity, and a CD4:CD8 ratio of 2:1. EBER‐ISH was positive in numerous cells, predominantly CD4‐positive/CD8‐negative, with a possible double‐negative subset and partial TIA‐1 expression. LMP was negative. Repeat EBV PCR demonstrated a decreased viral load (4052 IU/mL).

Collectively, recurrent photoprovoked ulcerative lesions, persistent EBV viraemia, reproducible EBER‐positive T cells in anatomically distinct biopsies and clonal T‐cell receptor gene rearrangement supported a diagnosis of classic HVLPD and argued strongly against a benign photodermatosis. Alternative EBV‐associated lymphoid neoplasms, including extranodal NK/T‐cell lymphoma, nasal type, were considered unlikely in the absence of histologic features of overt malignancy, CD56 expression or clinical evidence of systemic disease.

Next‐Generation Sequencing revealed a truncating variant in Krüppel‐like factor 2 (KLF2), which to our knowledge has not previously been described in HVLPD. A PET scan revealed no evidence of tracer‐avid lymphoma, supporting a cutaneous‐limited process. As of March 2026, the patient remains clinically stable under regular dermatologic surveillance with concurrent haematology review for early detection of systemic involvement. Current management comprises strict photoprotection, topical corticosteroids and aciclovir.

## 3 Discussion

Although MEDs were within normal limits, broadband UVA and solar simulator exposure reproducibly induced lesions consistent with the patient's habitual eruptions. This discordance suggests that photosensitivity in classic HVLPD may not manifest as reduced erythemal thresholds but rather as a disease‐specific UV‐provoked cutaneous response. Reliance on erythema‐based testing alone may therefore underestimate clinically relevant photosensitivity.

From a prognostic perspective, late‐adolescent onset is atypical and approaches the adult‐onset category, which has been associated with less favourable outcomes in some series [5]. However, despite persistent EBV viraemia, the sustained cutaneous‐limited and clinically indolent course observed in this case is consistent with reports describing a comparatively favourable trajectory in predominantly white cohorts [2]. These findings caution against drawing prognostic conclusions based solely on age at onset or viral load.

At the molecular level, KLF2 regulates lymphocyte quiescence and has been implicated in lymphoid malignancies through loss‐of‐function alterations [6]. Although no established role has been described in HVLPD, loss of KLF2‐mediated growth control could theoretically cooperate with EBV‐driven signalling to promote clonal T‐cell expansion, warranting further investigation within the HVLPD spectrum.

In summary, this case underscores the importance of maintaining clinical suspicion for lymphoproliferative disease in patients with persistent photosensitivity, even when initial presentations closely resemble benign photodermatoses. Although classic HVLPD is typically indolent, its placement within a spectrum necessitates ongoing surveillance due to the risk of systemic progression and lymphomatous transformation.

## Funding

The authors have nothing to report.

## Consent

Written informed consent was obtained for publication of this case and images.

## Conflicts of Interest

The authors declare no conflicts of interest.

## Data Availability

The data that support the findings of this study are available on request from the corresponding author. The data are not publicly available due to privacy or ethical restrictions.
